# Aspirin as a potential treatment in sepsis or acute respiratory distress syndrome

**DOI:** 10.1186/s13054-015-1091-6

**Published:** 2015-10-23

**Authors:** Philip Toner, Danny Francis McAuley, Murali Shyamsundar

**Affiliations:** Centre for Infection and Immunity, Queen’s University of Belfast, Health Sciences Building, Lisburn Road, Belfast, BT9 7AE Northern Ireland UK; Regional Intensive Care Unit, Royal Victoria Hospital, 274 Grosvenor Road, Belfast, BT12 6AB Northern Ireland UK

## Abstract

Sepsis is a common condition that is associated with significant morbidity, mortality and health-care cost. Pulmonary and non-pulmonary sepsis are common causes of the acute respiratory distress syndrome (ARDS). The mortality from ARDS remains high despite protective lung ventilation, and currently there are no specific pharmacotherapies to treat sepsis or ARDS. Sepsis and ARDS are characterised by activation of the inflammatory cascade. Although there is much focus on the study of the dysregulated inflammation and its suppression, the associated activation of the haemostatic system has been largely ignored until recently. There has been extensive interest in the role that platelet activation can have in the inflammatory response through induction, aggregation and activation of leucocytes and other platelets. Aspirin can modulate multiple pathogenic mechanisms implicated in the development of multiple organ dysfunction in sepsis and ARDS. This review will discuss the role of the platelet, the mechanisms of action of aspirin in sepsis and ARDS, and aspirin as a potential therapy in treating sepsis and ARDS.

## Introduction

The frequency with which people are admitted to hospital with sepsis or septic shock is rising, as is the proportion of cases who ultimately require admission to an intensive care unit (ICU) [1]. Sepsis accounts for 750,000 admissions per year in the USA, and the mortality is reported at approximately 30 % [2] and the estimated annual cost is $16.7 billion [2]. The acute respiratory distress syndrome (ARDS) is commonly associated with sepsis [3]. The annual incidence in the USA of ARDS is estimated at 190,600 [4], and mortality is currently at 25–35 % [5]. In addition to the impact on the patient, ARDS is estimated to cost approximately 3.6 million hospital days per year in the USA [4]. In those discharged from ICU following sepsis or ARDS, there is a significant functional impairment and decreased quality of life for several years [6]. Both conditions are associated with significant personal and financial burden to both families and society resulting from personal care costs and loss of employment.

Despite developments into the pathophysiology of sepsis and ARDS and advancements in prevention, treatment and education, there remains significant morbidity and mortality [7]. In sepsis, there is no specific pharmacotherapy [8]. Activated protein C, which was initially licensed for patients with severe sepsis, has been shown to have no demonstrable effect on mortality in sepsis and septic shock [9]. In ARDS, ventilation with a lung protective strategy has been shown to decrease mortality and increase ventilator-free days [10]. Early administration of cisatricurium improves adjusted 90-day mortality and increases ventilator-free days [11]. The mechanism for this benefit is still debated and may be due to improved ventilator synchrony leading to a reduction in ventilator associated injuries rather than as a specific ARDS therapy. In patients with ARDS, both conservative fluid management and corticosteroids have been shown to increase ventilator-free days [12, 13], although conservative fluid management is possibly associated with long-term cognitive dysfunction [14], and treatment with corticosteroids after 14 days is associated with an increase in mortality [13]. Multiple potential drug therapies, including most recently simvastatin [5], have been investigated, but as yet there is no known effective pharmacological treatment for ARDS [15, 16].

Derived from the myeloid line, megakaryocytes are the precursors to platelets. Once mature and fully differentiated, megakaryocytes form proto-platelet processes, which fragment off, forming the platelet. Platelets are anucleated cell fragments containing alpha granules, dense granules, and lysomes [17, 18]. These granules, when activated, release chemokines, prostaglandins and small molecules which promote a pro-inflammatory state and leucocyte migration [19]. There has been significant progress in our understanding of platelets in sepsis and in the complications of sepsis and ARDS [20]. The linking of thrombosis, inflammation, platelets and the possible therapeutic benefit of anti-platelet medications in sepsis and ARDS is an area in need of future research. In this review, we will look at the potential role of aspirin in the treatment of sepsis, septic shock and ARDS.

## Haemostatic and inflammatory cascade interplay

There is a close evolutionary association between the inflammatory cascade and the haemostatic system, and a single trigger mechanism of activation for both systems goes back 450 million years [21]. The common initiators for both pathways, such as endotoxin, highlight the complex interplay and overlap of these pathways. Historically, haemostasis formed a crucial part in the innate immune system where walling off of the pathogen was accomplished by the formation of fibrin, platelet and leucocyte clot which forms the basis of the “haemostatic containment” hypothesis [21]. Although this co-stimulation may have evolutionary advantages, severe sepsis is characterised by microvascular thrombosis which can contribute to multi-organ dysfunction (MOD) [22]. Interventions to reduce the haemostatic defect have been shown to improve organ function and reduce mortality in experimental models [23]. Similar changes in the pulmonary microcirculation are demonstrated in autopsy of lungs with ARDS [24]. An increase in pulmonary vascular dead space, which may reflect pulmonary microcirculation thrombosis, is associated with worse outcomes in patients with ARDS [25]. Platelet leucocyte interaction also enhances the production of inflammatory cytokines such as interleukin (IL)-1β, IL-8, monocyte chemotactic protein 1 and tumour necrosis factor alpha (TNFα), which propagates inflammation further.

## Platelets in sepsis and acute respiratory distress syndrome

Platelet activation by endotoxin and platelet-activating factors such as thrombin plays an important role in sepsis [26]. The sepsis and ARDS complications due to platelets are secondary to enhancement or dysregulation (or both) of their thrombotic and inflammatory actions [20, 27]. Once activated, the platelets alter shape, upregulate the expression of receptors like P-selectin, degranulate and aggregate [28]. This process promotes platelet adhesion with the endothelium, other platelets and leucocytes, leading to the formation and release of inflammatory and thrombotic agents, further leucocyte recruitment, oedema formation and production of neutrophil extracellular traps (NETs).

## Neutrophil extracellular traps

Intravascular NETs are protrusions of granulated chromatin with the purpose of capturing pathogens and result from the combination of activated neutrophils and platelets. Platelet interactions with the neutrophils are essential for their production as demonstrated by platelet depletion or disruption of the platelet neutrophil aggregation in mice [29]. Although the principle aim of NETs is entrapment of pathogens, over-production is associated with direct tissue and organ damage [30]. Furthermore, specifically in ARDS, the high concentration of pro-inflammatory factors in the alveoli can lead to excessive NET production, and the protrusions themselves can be a cause for direct mechanical injury to the lung tissue [30]. Recently, it has been reported that the NETs themselves can activate further platelets, promote fibrin deposition and act as supports for thrombosis formation, thus further perpetuating the inflammatory thrombotic process [31], ultimately resulting in MOD.

## Leucocyte recruitment and oedema formation

Platelets play a significant role in leucocyte recruitment, vascular permeability and resultant oedema formation. In a murine model of ARDS due to sepsis, platelet-depleted mice had reduced infiltration of neutrophils, reduced pulmonary oedema formation and better outcomes [32], which was felt to be secondary to the diminished leucocyte recruitment. Interestingly, platelet depletion resulted in significant reduction of pulmonary oedema in a transfusion-related model of acute lung injury while not influencing neutrophil migration [33]. Platelet depletion not only inhibited platelet activation and aggregation but also resulted in improved oxygenation, reduced pulmonary hypertension and less interstitial pulmonary oedema [34]. Furthermore, antagonising the effects of specific platelet-derived chemokines, namely CCL5 and CXCL4, reduces neutrophil migration, pulmonary oedema formation and tissue damage in the lungs [35].

These pre-clinical models highlight the importance of platelet activation in sepsis and ARDS and suggest that platelet depletion or inhibition of the platelet or platelet-specific chemokines can reduce platelet neutrophil aggregates and platelet sequestration and ultimately improve outcomes.

The evidence from pre-clinical research in sepsis is conflicting. In a murine model of *Staphylococcus aureus* septicaemia there was significantly increased bacterial burden, organ dysfunction and cytokine levels in platelet-depleted mice [36]. In addition, a *Klebsiella pneumoniae*-driven sepsis model, with significant platelet depletion, was associated with worse mortality and haemorrhage at the primary site of infection but with no influence on neutrophil recruitment to the lungs [37].

## Clinical significance of platelet activation in sepsis and acute respiratory distress syndrome

Activated platelets are found in significant quantities in the organs of patients with sepsis and septic shock [38]. A study in critically ill patients with sepsis concluded that this enhanced and uncontrolled adhesion of the platelets to leucocytes and the endothelium leads to their accumulation in the micro-circulatory system and eventual thrombosis formation contributing to MOD [39] in sepsis [22].

Platelets have been shown to accumulate in the lungs of patients with ARDS. In fact, platelet activation, migration and accumulation in the alveoli are major features of ARDS. Typically, bronchoalveolar lavage fluid from patients with ARDS has excessive concentrations of platelet-specific alpha granule proteins, suggesting high platelet activity [40], and following initial injury, leucocyte platelet aggregates form and increase dramatically in the alveolar tissue [41]. This enhanced and unregulated platelet activity leads to increasing leucocyte concentrations in the alveolar tissue and ultimately lung tissue damage. This was confirmed in lung biopsies from patients with diffuse alveolar damage which were found to have an exaggerated number of leucocytes within the small airways because of excessive platelet activation [42].

## Rationale for aspirin in sepsis and acute respiratory distress syndrome

Initially used by the ancient Greeks in the form of willow leaf tea and later refined in Germany in the 19th century, by Felix Hoffman, aspirin has become one of the most commonly used drugs today [43]. Aspirin is a non-selective inhibitor of the enzyme cyclooxygenase (COX), has a half-life of approximately 20 minutes and is subject to significant first-pass metabolism, and most of its action occurs in the portal circulation of the liver [43]. Aspirin has previously been used in high doses for the treatment of rheumatic fever, but currently low-dose aspirin continues to be used in both primary and secondary prevention in cardiovascular medicine.

**Fig. 1:**
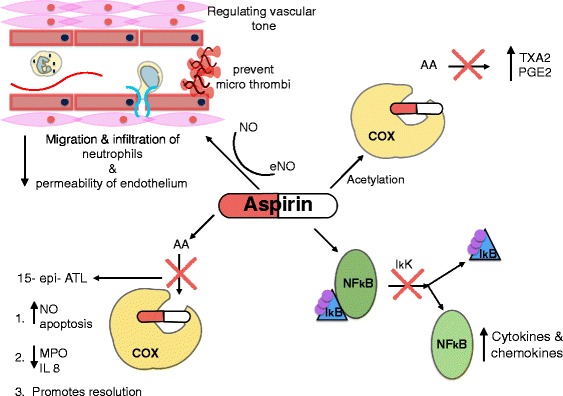
Mechanisms in which aspirin can manipulate the process in sepsis and acute respiratory distress syndrome: Inhibit the enzyme COX, preventing the formation of pro-inflammatory thromboxane and prostaglandins. Inhibit the release of NFκB from its inhibitor IkB, preventing the formation of pro-inflammatory cytokines and chemokines. Production of aspirin triggered lipoxin, which induces the release of NO, inhibits production of IL-8 and MPO, restores neutrophil apoptosis and promotes resolution. Increase production of NO, resulting in reduced migration and infiltration of neutrophils and reduced permeability of endothelium. *15-epi-ATL*, aspirin-triggered 15-epi-lipoxin A4, *AA* arachidonic acid, *COX* cyclooxygenase, *eNO* endothelial nitric oxide, *IKK* IkB kinase, *IL-8* interleukin 8, *MPO* myeloperoxidase, *NFκB* nuclear factor kappa B, *NO* nitric oxide, *PGE*_*2*_ prostaglandin E_2_, *TXA*_*2*_ thromboxane

There are several mechanisms in which aspirin can manipulate the processes involved in both sepsis and ARDS (Fig. 1): 1) inhibition of COX [43]; 2) inhibition of nuclear factor kappa B (NFκB) [44]; 3) production of nitric oxide (NO) [45]; and 4) lipoxin production [46].

### Inhibition of cyclooxygenase

The most obvious mechanism is irreversible inhibition of both COX I and COX II enzymes [43]. The inhibition results from the direct acetylation and obstruction of the active portion of the enzyme, thus preventing interaction with the substrate. This inhibition prevents the conversion of membrane phospholipid-derived arachidonic acid to thromboxane (TXA_2_) and prostaglandins, including the pro-inflammatory prostaglandin E_2_ (PGE_2_) [47]. As the platelet is anucleated, it has limited ability to replicate new proteins or enzymes, thus resulting in irreversible inhibition of the enzyme for the life span of the platelet, namely 7–10 days. Aspirin is significantly more potent at inhibiting COX I, especially at the lower 75 mg dose, than COX II. COX I is responsible for normal haemostatic processes, including platelet activation and aggregation through TXA_2_ production, which is a feature of both sepsis and ARDS [41]. COX II undergoes increased expression following stimulation from IL-1, TNFα and lipopolysaccharide (LPS) and results in increased production of prostaglandins, including PGE_2_ [48]. PGE_2_ is required for the production of pro-inflammatory cytokines and mediates the formation of oedema [49].

### Inhibition of nuclear factor kappa B production

As well as direct inhibition of COX, aspirin has been shown to downregulate the production of pro-inflammatory cytokines. NFκB is an important transcription factor required for production of pro-inflammatory interleukins and cytokines. Aspirin prevented NFκB production and ultimately leucocyte adhesion in a stimulated human epithelial cell model [44] and it does this by preventing the release of NFκB from its cytosolic inhibitor IκBa [50]. However, this specific effect was demonstrated only after treatment with high-dose aspirin of 10 or 20 mM, which is higher than the serum therapeutic concentration. Inflammation leads to an acidic environment and an acidic extracellular interstitial environment. This can enhance salicylate accumulation [51] because of local ion trapping and lead to higher local concentrations than serum concentrations.

### Production of nitric oxide

Low-dose aspirin reduced inflammation within the vascular endothelium and led to the development of smaller atherosclerotic lesions with less macrophages in low-density lipoprotein receptor-deficient mice [52]. In a study using dissected porcine coronary arteries, aspirin was shown to directly acetylate the endothelial nitric oxide synthase protein, thus releasing NO from the coronary artery endothelium. NO acts as an anti-adhesive, inhibiting the migration and infiltration of leucocytes through the endothelium as well as regulating vascular tone and micro-thrombi formation in the septic state [53]. Importantly, this was independent of COX inhibition as demonstrated by a lack of effect with indomethacin, another non-steroidal anti-inflammatory drug (NSAID), or with an aspirin metabolite [45].

### Lipoxin production

Recent evidence has also demonstrated anti-inflammatory properties with aspirin not seen in other NSAIDs. Aspirin can induce the production of a type of lipoxin called aspirin-triggered 15-epi-lipoxin A4 (ATL) [46] and can do so at the lower 75 mg dose [54]. Once the active site of the COX enzyme is blocked by the acetylation action of aspirin, the arachidonic acid is converted to ATL via 15-R-hydroxyeicosatetraenoic acid [55]. The anti-inflammatory effects of ATL have been extensively demonstrated in the pre-clinical septic models and LPS models of ARDS. ATL inhibits the production of IL-8 through inhibition of NFκB, thus reducing inflammation and leucocyte migration [56], and can independently trigger the release of NO [49]. ATL suppresses the anti-apoptotic effects of myeloperoxidase via inhibition of the B2 integrin signalling pathway, thus restoring the natural cell cycle of the polymorphonuclear neutrophils (PMNs), leading to effective resolution of inflammation [46, 57]. In addition, lipoxins can stimulate phagocytosis of apoptotic neutrophils by macrophages, possibly through enhancement of macrophage neutrophil adhesions permitting efficient resolution of inflammation [58]. Persistent inflammation and delayed apoptosis of PMNs are features of ARDS and are associated with worse outcomes [59].

Furthermore, in two experimental murine models of ARDS, one with intra-tracheal LPS and the second a transfusion-related acute lung injury, ATL significantly reduced the concentration of neutrophil platelet aggregates via antagonism of the lipoxin A4 receptor, resulting in decreased neutrophil migration, pulmonary oedema and vascular permeability [41]. Finally, ATL significantly improved 48-hour survival and decreased BAL concentrations of TNFα and macrophage inflammatory protein-2 following LPS-induced lung injury in mice [60].

## Pre-clinical evidence for aspirin in sepsis and acute respiratory distress syndrome in animal models

Aspirin has been shown to be effective in murine models of sepsis and ARDS. Mice injected with *Salmonella enteritidis* endotoxin were pre-treated with aspirin 30 minutes prior to infection at varying dosages. A significant 24-hour survival rate benefit was demonstrated with 3.75, 15 and 30 mg/kg of aspirin [61].

Zarbock et al. confirmed that platelet neutrophil aggregates are a significant feature in ARDS and demonstrated that inhibition of this aggregation with 1 mg/g aspirin reduced neutrophil recruitment and improved gas exchange and survival in a mouse model of ARDS induced by intra-tracheal hydrochloric acid [62]. In support of this, a two-hit model of ARDS induced in mice that were exposed to LPS for 24 hours, then injected with major histocompatibility complex 1 mAB and were either depleted of platelets or pre-treated with aspirin 100 μg/g intraperitoneally found that both interventions reduced lung injury and mortality [63]. It should be noted, however, that the dose used would equate to a relatively large therapeutic dose in humans. Interestingly, Grommes et al. [35] and Looney et al. [63] both found that blockage of the P selectin receptor or glycoprotein IIb/IIIa receptors on the platelet did not confer the same benefits, possibly suggesting that the outcomes are not simply related to platelet neutrophil aggregation but that aspirin itself may confer additional benefits.

## Pre-clinical human evidence for aspirin in sepsis and acute respiratory distress syndrome

Leucocyte infiltration and oedema formation in a cantharidin-generated dermal blister was assessed in healthy male subjects. Initially, the blisters were induced on a forearm, without aspirin, and assessed over the course of 72 hours for inflammatory response, cell count and period of resolution. In a crossover study, the same volunteers had blisters induced on the other forearm following a 10-day course of 75 mg aspirin. The study concluded that low-dose aspirin, through the production of ATL and NO, significantly reduced the accumulation of macrophages and neutrophils at the site of the blister but had no effect on oedema formation [49].

There are no published studies of aspirin in human pre-clinical models of ARDS. There is ongoing work in our group to study the effects of aspirin in a human *ex vivo* lung perfusion model of ARDS and in an inhaled LPS model in healthy human volunteers (NCT01659307).

## Observational evidence for aspirin in sepsis and acute respiratory distress syndrome

Supporting the pre-clinical studies, several observational studies have assessed a possible association with pre-hospital anti-platelet (the majority of which is aspirin) therapy and sepsis or ARDS. These are almost exclusively single-centre retrospective observational cohort studies and range from over 600,000 patients [64] to 22 patients [65] (Table 1). Patients admitted with community-acquired pneumonia on anti-platelet therapy have a lower admission rate to the ICU and shorter hospital stay [66]. In a general population of ICU admissions, those on anti-platelet therapy have a decreased mortality [66, 67] and have a decreased risk of developing ARDS [68–70] and multi-organ failure [71]. Furthermore, in those ICU patients with septic shock or ARDS being treated with anti-platelet therapy, there was a reduction in mortality [64, 66, 72–74]. Finally, in a prospective cohort study of patients admitted to a medical or surgical ICU investigating pre-hospital statin and aspirin therapy and the development of sepsis or ARDS and mortality in ICU patients, aspirin alone did not significantly affect the development of sepsis or ARDS, but there was a trend toward reduced mortality. In addition, the group prescribed the combination of aspirin and a statin had the lowest rate of sepsis or ARDS; however, this was not statistically significant [75].

**Table 1:** A summary of recent cohort studies into anti-platelet therapy and sepsis/acute respiratory distress syndrome

Reference	Design	Date	Country	Sample size	Investigating	Results
[69]	Retrospective observational cohort	2006	USA	161 (68 on ASA 11 other)	Association of pre-hospital APT and risk of ARDS in the critically ill	Decreased incidence of ARDS in APT group but no change in mortality or number of ICU days
[66]	Three retrospective observational cohorts	2011	Germany	1) 224	1) Association of pre-hospital APT and outcome of patients with community-acquired pneumonia	1) Decreased ICU admission and hospital stay with APT
				2) 615	2) Association of pre-hospital APT and outcome of ICU admissions within 24 hours of hospitalisation	2) Decreased mortality with APT group
				3) 834	3) Association of pre-hospital APT mortality in critically ill patients with sepsis or septic shock	3) Decreased ICU mortality with the APT group
[72]	Retrospective cohort study	2000–2009	Australia	5523	Association of ASA in SIRS or septic shock and mortality	Significant improvement in mortality in ASA group
[68]	Historical cohort study	2007–2009	USA	651	Association of pre-hospital ASA and mortality, risk of ARDS, development of septic shock and length of stay in critically ill	No association between pre-hospital ASA use and mortality but reduction in ARDS and decrease in ventilator-free days
[76]	Secondary analysis of prospective multi-centre international cohort study	2011	20 sites in USA 2 sites in Turkey	3855	Association of pre-hospital APT and risk of ARDS	No significant reduction in ARDS in the ASA group
[75]	Prospective cohort study	2006–2008	USA	575	Association of pre-hospital APT and risk of ARDS and septic shock in the critically ill	No difference in ARDS or septic shock development with pre-hospital statins or ASA
[67]	Retrospective cohort	2010	Germany	615	Association of pre-hospital APT mortality in critically ill	Reduction in mortality in APT group
[74]	Prospective observational cohort analysis	2010–2012	UK	202	Association of pre-hospital APT and mortality in ARDS	Reduction in mortality in ASA group
[83]	Retrospective cohort study	2013	Germany	886	Association of pre-hospital APT and mortality in critically ill	Significant reduction in mortality with ASA; there was no additional benefit from adding clopidogrel
[70]	Secondary analysis of prospective study	2006–2012	USA	1149	Association of pre-hospital APT and risk of ARDS in critically ill	Reduction in mortality with ASA group
[71]	Secondary analysis of cohort study	2001–2008	USA	839	Association of pre-hospital APT and risk of MOF, lung dysfunction and mortality in trauma patients	Reduction in risk of lung dysfunction, MOF and possible mortality in trauma patients who received a blood transfusion in APT group
[65]	Retrospective single-centre study	2008–2013	USA	22	Association of pre-hospital APT and risk of ARDS in patients with post-aortic valve replacement surgery	No association between ASA and incidence of ARDS in patients with post-aortic valve replacement surgery
[64]	Observational cohort study	2000–2010	Taiwan	683,421	Association of pre-hospital APT and mortality in sepsis	Reduction in mortality in APT group

*ASA* aspirin, *APT* anti-platelet therapy, *ARDS* acute respiratory distress syndrome, *ICU* intensive care unit, *SIRS* systemic inflammatory response syndrome, *MOF* multi-organ failure

Not all the observational studies found a correlation between anti-platelet therapy and outcomes in sepsis and ARDS. A multi-centre analysis of association between pre-hospital aspirin use and development of ARDS across the USA and Turkey found no significant association [76]. This study involved 20 American hospitals and two hospitals in Turkey; this imbalance makes it difficult to adjust for confounding factors and differences in treatment protocols. Also, a study looking at the incidence of ARDS in patients with post-aortic valve replacement concluded that there was no significant difference between those admitted on aspirin and those not; however, the population was small (22 patients) [65].

It is important to highlight the limitations of observational studies. Owing to the observational nature of the study design, there is no control over the administration, dose and compliance to treatment. There could be further confounding factors such as access to health care, in that patients prescribed the medication may have better management of their chronic conditions or the fact that medications including aspirin may be discontinued in the sickest of the critically ill cohort.

## Randomised controlled trials for sepsis and acute respiratory distress syndrome

There is a paucity of randomised clinical trials investigating aspirin in sepsis or ARDS. There is one large randomised control trial examining the role of NSAIDs, specifically intravenous ibuprofen for 48 hours, in critically ill patients with sepsis. This study included 455 patients and concluded that ibuprofen reduced levels of pyrexia and tachycardia but did not prevent the development of septic shock or ARDS or improve mortality [77]. Because aspirin, unlike other NSAIDs, can exert COX-independent effects, additional studies evaluating the benefits of aspirin are needed.

## Challenges of aspirin usage in the critically ill

Sepsis and ARDS are commonly associated with thrombocytopenia. It is seen in 20–40 % of critically ill patients [78], but the incidence varies depending on the cutoff used to define thrombocytopenia. In a study of patients with thrombocytopenia in the ICU, the risk of any type of bleeding increased from 4.1 % in patients without thrombocytopenia to approximately 53 % in patients with a platelet count of less than 100 × 10^9^/l [79]. Bleeding was described as major (life-threatening, compromising haemodynamic status or requiring urgent intervention), moderate (requiring non-urgent transfusions) or minor (all other events). There were 29 major, 21 moderate and 11 minor episodes of bleeding in this cohort. Thrombocytopenia was deemed as causative/contributory to 23 events out of these 61 events. There was no analysis comparing the severity of the bleeding with the platelet count. More recently, a retrospective analysis of thrombocytopenia in mixed ICU admissions concluded that there is no significant association between 28-day mortality and thrombocytopenia, defined as a platelet count of less than 150 × 10^9^, but the patients with thrombocytopenia had an increased incidence of major bleeding at 14.4 % compared with 3.7 % [80]. Major bleeding, however, was defined as intracranial or retroperitoneal or as any overt bleeding or a fall in haemoglobin greater than 2 g/dl. Although thrombocytopenia was defined, the incidence of major bleeding (or the individual components of this composite measure) is not reported according to the degree of severity of thrombocytopenia. Furthermore, in patients admitted to the ICU with community-acquired pneumonia, only a platelet count of less than 50 × 10^9^/l was associated with a significant increase in mortality.

The safety of aspirin in patients with thrombocytopenia has not been studied in critically ill patients in a prospective randomised controlled trial. However, in a retrospective cohort study of patients in a mixed ICU, there was a mortality benefit from anti-platelet drugs irrespective of their bleeding risk. Bleeding was defined as any incident described as ‘bleeding’ in the clinical notes [67]. In a non-ICU population, there was no increased risk of bleeding in cancer patients who received aspirin in the setting of acute coronary syndrome, even in patients with a platelet count of less than 100,000/μl [81]; in this study, the median platelet count in the thrombocytopenic group was 32 × 10^9^/l and the range was 4 to 100 × 10^9^/l. These studies suggest the benefit of aspirin even in patients with thrombocytopenia, but caution is needed given the limits of the observational design of these studies.

## Future direction

Several clinical trials are currently exploring aspirin both as a preventative agent and as a treatment in sepsis and ARDS. In Australia, the ANTISEPSIS trial (Aspirin to Inhibit Sepsis, ACTRN12613000349741) will assess whether 100 mg of aspirin daily for 5–7 years reduces severity of sepsis by preventing admissions to the hospital or ICU and improving mortality. In Brazil, Aspirin for the Treatment of Sepsis (NCT01784159) is a phase 2 trial investigating the effect of 200 mg of aspirin on organ dysfunction, Sequential Organ Failure Assessment score and duration of ventilation in patients with sepsis. The US Critical Illness and Injury Trials Group (USCIITG) is conducting a multi-centre, double-blind, randomised control trial testing the hypothesis that early treatment with aspirin will prevent ARDS (Lung Injury Prevention Study With Aspirin (LIPS-A)) [82]. Patients were randomly assigned to receive either placebo or an initial dose of 325 mg of aspirin followed by 7 days of 81 mg, with the primary outcome being the development of ARDS. The study has completed recruitment. Finally, in the UK, a randomised, double-blind, allocation-concealed, placebo-controlled phase 2 single-centre trial of aspirin as a treatment for ARDS (aSpirin as a Treatment for ARDS, STAR Trial, NCT02326350) has recently started. Patients, within 72 hours of a diagnosis of ARDS, will be randomly assigned to either aspirin 75 mg or placebo once daily for a maximum of 14 days. The primary outcome measure is oxygenation index at day 7.

## Conclusions

Despite the advancements in knowledge of the pathophysiology in sepsis and ARDS, there remains a significant human and economic impact on society as a whole, and there is no effective pharmacological treatment for ARDS. There has been and continues to be extensive work on the role platelets play in sepsis and ARDS. It has been demonstrated through laboratory in vitro studies, animal studies, and observational analysis that aspirin may be of benefit in the treatment of sepsis and ARDS. The results of ongoing randomised controlled trials will help elucidate the role of aspirin in treating sepsis and ARDS.

## Abbreviations

ALT: Aspirin-triggered 15-epi-lipoxin A4
ARDS: Acute respiratory distress syndrome
COX: Cyclooxygenase
ICU: Intensive care unit
IL: Interleukin
LPS: Lipopolysaccharide
MOD: Multiple organ dysfunction
NET: Neutrophil extracellular trap
NFκB: Nuclear factor kappa B
NO: Nitric oxide
NSAID: Non-steroidal anti-inflammatory drug
PGE_2_: Prostaglandin E_2_
PMN: Polymorphonuclear neutrophil
TNFα: Tumour necrosis factor alpha
TXA_2_: Thromboxane
